# Detection of pediatric drug-induced kidney injury signals using a hospital electronic medical record database

**DOI:** 10.3389/fphar.2022.957980

**Published:** 2022-09-23

**Authors:** Yuncui Yu, Xiaolu Nie, Yiming Zhao, Wang Cao, Yuefeng Xie, Xiaoxia Peng, Xiaoling Wang

**Affiliations:** ^1^ Department of Pharmacy, National Center for Children’s Health, Beijing Children’s Hospital, Capital Medical University, Beijing, China; ^2^ Clinical Research Center, National Center for Children’s Health, Beijing Children’s Hospital, Capital Medical University, Beijing, China; ^3^ Center for Clinical Epidemiology and Evidence-Based Medicine, National Center for Children’s Health, Beijing Children’s Hospital, Capital Medical University, Beijing, China; ^4^ Information Center, National Center for Children’s Health, Beijing Children’s Hospital, Capital Medical University, Beijing, China

**Keywords:** drug-induced kidney injury, children, active monitoring, electronic health records, signal detection

## Abstract

**Background:** Drug-induced kidney injury (DIKI) is one of the most common complications in clinical practice. Detection signals through post-marketing approaches are of great value in preventing DIKI in pediatric patients. This study aimed to propose a quantitative algorithm to detect DIKI signals in children using an electronic health record (EHR) database.

**Methods:** In this study, 12 years of medical data collected from a constructed data warehouse were analyzed, which contained 575,965 records of inpatients from 1 January 2009 to 31 December 2020. Eligible participants included inpatients aged 28 days to 18 years old. A two-stage procedure was adopted to detect DIKI signals: 1) stage 1: the suspected drugs potentially associated with DIKI were screened by calculating the crude incidence of DIKI events; and 2) stage 2: the associations between suspected drugs and DIKI were identified in the propensity score-matched retrospective cohorts. Unconditional logistic regression was used to analyze the difference in the incidence of DIKI events and to estimate the odds ratio (OR) and 95% confidence interval (CI). Potentially new signals were distinguished from already known associations concerning DIKI by manually reviewing the published literature and drug instructions.

**Results:** Nine suspected drugs were initially screened from a total of 652 drugs. Six drugs, including diazepam (OR = 1.61, 95%CI: 1.43–1.80), omeprazole (OR = 1.35, 95%CI: 1.17–1.54), ondansetron (OR = 1.49, 95%CI: 1.36–1.63), methotrexate (OR = 1.36, 95%CI: 1.25–1.47), creatine phosphate sodium (OR = 1.13, 95%CI: 1.05–1.22), and cytarabine (OR = 1.17, 95%CI: 1.06–1.28), were demonstrated to be associated with DIKI as positive signals. The remaining three drugs, including vitamin K1 (OR = 1.06, 95%CI: 0.89–1.27), cefamandole (OR = 1.07, 95%CI: 0.94–1.21), and ibuprofen (OR = 1.01, 95%CI: 0.94–1.09), were found not to be associated with DIKI. Of these, creatine phosphate sodium was considered to be a possible new DIKI signal as it had not been reported in both adults and children previously. Moreover, three other drugs, namely, diazepam, omeprazole, and ondansetron, were shown to be new potential signals in pediatrics.

**Conclusion:** A two-step quantitative procedure to actively explore DIKI signals using real-world data (RWD) was developed. Our findings highlight the potential of EHRs to complement traditional spontaneous reporting systems (SRS) for drug safety signal detection in a pediatric setting.

## 1 Introduction

Drug-induced kidney injury (DIKI), as one of the most common adverse drug reactions (ADRs), is a significantly increased clinical problem worldwide. It may lead to clinical symptoms such as oliguria, anuria, and acute renal failure (Radi, 2019). Studies have shown that the incidence of DIKI in hospitalized patients is about 2%–5%, accounting for 19%–40% of acute kidney injury (AKI) (Hosohata, 2016). Children are at higher risk of developing DIKI compared with adults; several factors contribute to the pediatric renal damage onset, including the immaturity of renal functions and specific pathological conditions (Faught et al., 2015a). Data from critically ill children who have DIKI suggest that survivors develop a risk for the development of chronic kidney disease (Hanna et al., 2016). In addition to that, DIKI is more likely to occur in the post-marketing of drugs than in the pre-marketing setting as its incidence is low (Faught et al., 2015b). This is particularly true for children since they are not frequently included in clinical trials. It is of great value to prevent and reduce DIKI in pediatrics through a post-marketing approach.

In most countries all over the world, national pharmacovigilance relies heavily on spontaneous reporting systems (SRS), in which suspected ADRs are reported to a national coordinating center by health professionals, manufacturers, or directly by patients. However, as a passive monitoring method, SRS has its inherent limitations, such as poor-quality reports, underreporting, and the inability to estimate rates and frequencies of ADRs (Pitts and Le Louet, 2018). Previous studies have demonstrated that 50%–90% of DIKI were unreported using SRS (Yang et al., 2015). Additional resources and methods are, therefore, needed to actively monitor the quantitative aspects of drug safety and to characterize ADRs associated with specific drugs and in specific populations. Recently, real-world data (RWD), especially data collected during routine clinical care in the form of electronic health records (EHRs), have been adopted by healthcare professionals, regulators, and providers to inform decision-making about drug safety. Several emerging pharmacovigilance programs extend the pharmacovigilance capabilities by monitoring drug safety signals actively using EHRs, such as the “Sentinel Initiative” in the United States (Toh et al., 2016), the “Exploring and Understanding Adverse Drug Reaction Project (EU-ADR)" in Europe (de Bie et al., 2015), and the “Adverse Drug Events Active Surveillance and Assessment System (ADE-ASAS)” in China (Chen et al., 2020). More attempts have also been made to develop data-mining methods based on EHRs, such as the “Global Trigger Tool (GTT)” (Garrett et al., 2013) and the “Comparison of the Laboratory Extreme Abnormality Ratio (CLEAR) algorithm” (Yoon et al., 2012). However, these methods are not applicable for the detection of DIKI signals because the confounding factors affecting DIKI are not fully considered. Moreover, most of these studies are conducted on adults, and so far, little is known about pediatric patients in relation to this issue.

Hence, the present study aims at developing a novel quantitative algorithm for DIKI signal detection using a hospital electronic medical record database and analyzing the correlation and characteristics of DIKI signals with the specific drugs in the pediatric population.

## 2 Methods

### 2.1 Data sources

This study was conducted using the retrospective inpatient data warehouse of Beijing Children’s Hospital (BCH) from 1 January 2009 to 31 December 2020 (Yu et al., 2020). Approximately, 575,965 records of inpatients under 18 years old were included along with their detailed diagnoses, medications, and laboratory tests. Considering the immature kidney function in the neonates, only inpatients aged 28 days to 18 years old were included. All eligible patient data were exported and de-identified to protect their privacy.

The protocol of the study was approved by the Institutional Review Board (IRB) of BCH, Capital Medical University (approval number: 2018-129), with a waiver of informed consent. This work was reported critically according to the RECORD statement for pharmacoepidemiology (RECORD-PE) statement (Supplementary Table S1).

### 2.2 Laboratory criterion of DIKI

According to the published Kidney Disease: Improving Global Outcomes (KDIGO) (Ostermann et al., 2020), the serum creatinine (SCr) and the glomerular filtration rate (GFR) were the most important renal function parameters for determining DIKI. Because of the difficulty in measuring directly in clinical settings, the estimated GFR (eGFR) was usually used instead of the GFR and was calculated using the following formula, based on Scr and the normalization constant (Q) (Hoste et al., 2014):

$$eGFR=107.3\times (1-e^{-Age/0.5})/\left(SCr/Q\right).$$

(For children, Q is the median or the average Scr concentration for healthy children and depends linearly on age)

The fourth-degree polynomials for Q are as follows:

$$Q=17.8+6.68\times Age-0.907\times {Age}^{2}+0.0687\times {Age}^{3}-0.00152\times {Age}^{4}\left(\text{boys}\right)$$

$$Q=18.2+5.54\times Age-0.602\times {Age}^{2}+0.0421\times {Age}^{3}-0.00993\times {Age}^{4}\left(\text{girls}\right)$$

After completing the calculation, the normal pediatric reference interval of the eGFR was as follows: 33–84(3–6 months), 57–122(6–12 months), 78–132(12–15 months), 84–150(15–24 months), and 83–143 (24 months–18 years old).

Thus, according to the KDIGO guidelines and the reference interval of pediatric kidney parameters (Zhu, 2015), the trigger for pediatric DIKI was defined as the following events that occurred after medication within the appropriate therapeutic dose range: (1):SCr>130 mmol/L; or (2) out of the reference interval of the eGFR for pediatrics.

### 2.3 Establishment of the two-stage signal detection model for DIKI

#### 2.3.1 Stage 1: Screening suspected drugs

**FIGURE 1:**
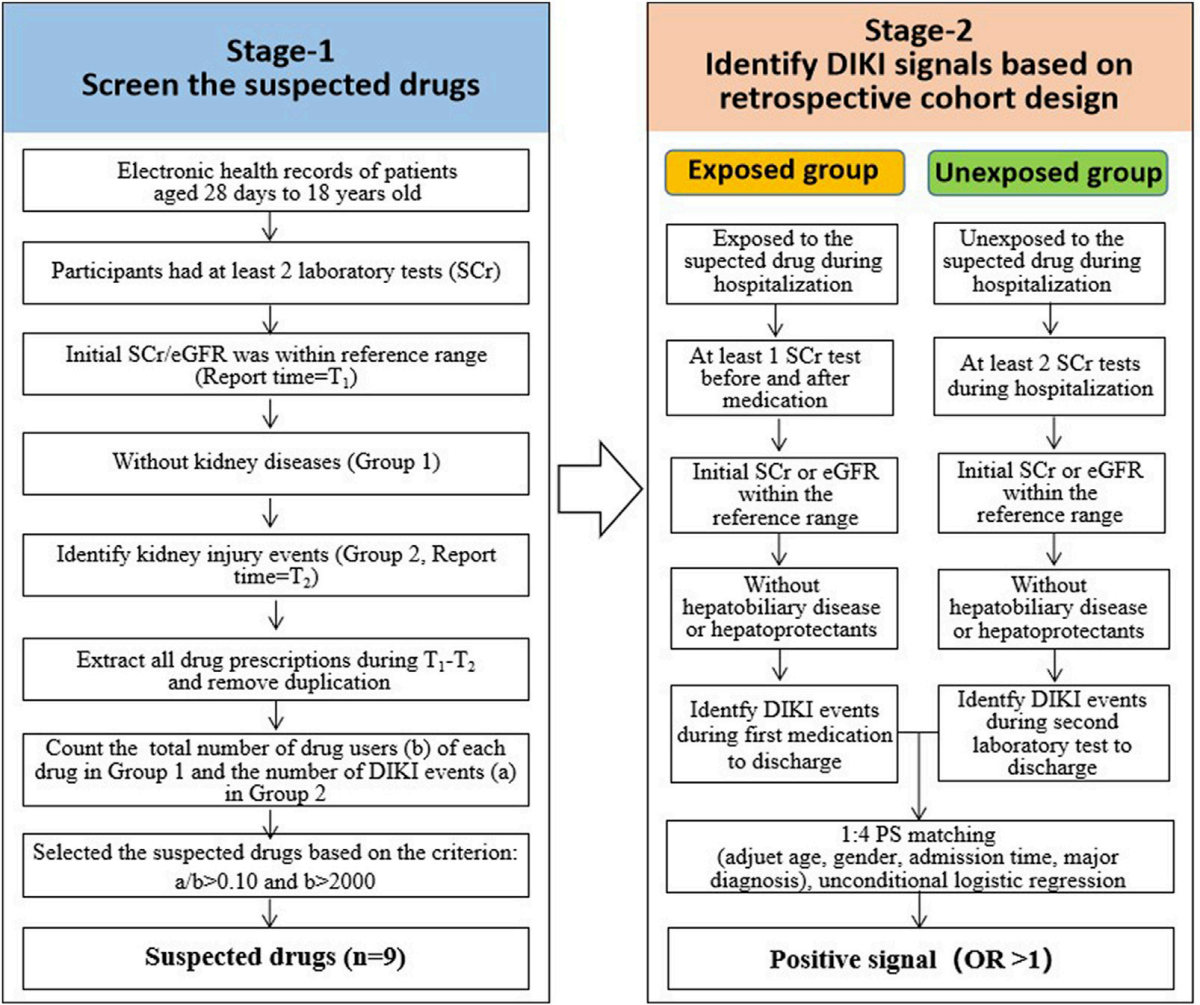
Workflow for identifying potential DIKI signals based on the retrospective cohort design. Abbreviations: DIKI: drug-induced kidney injury; EHRs: electronic health records. SCr: serum creatinine; eGFR: estimate glomerular filtration rate; OR: odds ratio.

The main purpose of this step was to identify the suspected drugs that may cause DIKI to provide candidate drugs for the subsequent correlation analysis. The main steps were as follows (Figure 1):

1) The records with at least two SCr tests in the eligible participants were selected.
2) The records with the initial SCr or eGFR value within the reference range were further included. The report time of SCr tests was recorded as T1. This step aimed to ensure that kidney function was normal before medication.
3) The records with the diagnosis of kidney-related diseases were excluded (Supplementary Table S2). This step aimed to exclude the records where changes in laboratory parameters were primarily due to the progression of the kidney-related disease itself, rather than DIKI. The remaining records were marked as group 1.
4) The records with abnormal SCr or eGFR test findings, which were considered as DIKI events, were marked as group 2. The time of the first abnormal result was recorded as T2.
5) All drugs during T1-T2 were extracted, and duplicates were removed from groups 1 and 2. Thus, the number of DIKI events (a) and the total number of drug users (b) of each drug were obtained.
6) We calculated the ratio of a/b for each drug. The threshold of the a/b ratio was set according to the range of a/b values of solvents for intravenous infusions, such as normal saline and glucose injections, which can be regarded as the reference value since it is well known that they do not affect DIKI. The a/b values of solvents for intravenous infusions ranged from 0.081 to 0.095. In addition to that, for drugs with less than 2000 users, the number of DIKI events in the exposed group was too low. There may be a greater risk of bias in the subsequent statistical analysis due to insufficient samples. Thus, the criteria for suspected drugs were as follows: (1): ratio (a/b) > 0.10 and (2) b > 2000.

#### 2.3.2 Stage 2: Identifying potential DIKI signals based on the retrospective cohort design

This step aimed to compare the incidence of DIKI events between the exposed group (i.e., taking the suspected drugs) and the unexposed group (i.e., not taking the suspected drugs) whilst also adjusting for confounding factors through retrospective analysis. This was performed to explore the association between drugs and kidney damage. The following analysis was performed on all suspected drugs found in stage 1 (Figure 1):

##### 2.3.2.1 Exposure group

1) All records containing suspected drugs were identified.
2) The records with at least one SCr test before and after medication were screened.
3) The records with an initial SCr or eGFR result that was within the normal range before the first medication were included.
4) The records with competing kidney diseases were excluded (Supplementary Table S2).
5) In cases where records showing abnormal SCr or eGFR findings during hospitalization, the records with kidney-protecting drugs before the first report time of the abnormal test were excluded (Supplementary Table S3); for records without SCr or eGFR abnormalities, those who have used kidney-protecting drugs during the entire hospitalization were excluded.

##### 2.3.2.2 Unexposed group

1) All records without the suspected drug were identified.
2) The records with at least two SCr tests during hospitalization remained.
3) The records with an initial SCr or eGFR that was within the normal range after admission were included.
4) The records with competing kidney diseases were excluded (Supplementary Table S2).
5) In cases where records showing abnormal SCr or eGFR findings during hospitalization, the records with kidney-protecting drugs before the first report time of the abnormal test were excluded (Supplementary Table S3); for records without SCr or eGFR abnormalities, those who have used kidney-protecting drugs during the entire hospitalization were excluded.

#### 2.3.3 Signal detection

1) Each exposed record was paired with four unexposed records randomly after adjusting age, gender, admission time, and major diagnosis.
2) The association between suspected drugs and kidney injury was analyzed by unconditional logistic regression. The odds ratio (OR) and 95% confidence interval (CI) were calculated.
3) An OR>1.0 (with the 95%CI lower band >1) indicated a positive signal; otherwise, a negative signal was considered.

### 2.4 Evaluation of the DIKI signals

A literature search and the summary of product characteristics (SPCs) were used as available knowledge to evaluate the novelty of the positive DIKI signals. Type I signals were defined as those signals that have not been previously documented in research in both children and adults. Type II signals were defined as those drug–DIKI associations that have not been reported in children, although they have been reported in adults. The SPCs were checked from the FDA website (https://www.fda.gov), Micromedex (https://www.ibm.com/watson-health/learn/micromedex), and the drug instructions (https://www.yaozh.com/). Literature research was conducted using PubMed (https://pubmed.ncbi.nlm.nih.gov), Embase (https://www.embase.com), Wanfang (http://www.wanfang.data.com.cn/index.html), and CNKI (http://www. cnki.net/).

### 2.5 Statistical analysis

MySQL software version 14.14 (Oracle, California, United States) was used as a database management system. The pandas v1.2.2 package in Python 3.7 was performed to summarize data. R 4.2.0 software was used for statistical analysis. The forest plot was visualized by GraphPad Prism 9.3 software.

The propensity score matching (PSM) was used to match the exposed group and unexposed group for the ratio of 1:4. As demographic variables, the distribution of age and gender needs to be comparable in exposed and unexposed groups. Laboratory testing equipment, methods, and capability may change over time in the hospital, which can affect the results of laboratory parameters such as Scr. The patients admitted at similar times in the two groups were, therefore, more comparable. In addition, as the patient’s underlying medical condition may affect the effectiveness of the drugs on renal function, cases with similar diagnoses in two groups need to be matched. Thus, four variables, namely, age, gender, admission time, and main diagnosis, were considered as confounding factors. The logistic regression model was used to calculate propensity scores, with drug exposure or not as dependent variables and four confounding factors as covariates. The nearest neighbor matching principle was used with a caliper of 0.1.

An unconditional logistic regression was used to analyze the association between suspected drugs and kidney injury. All *p*-values were reported as two-sided, and *p* < 0.05 represented statistical significance.

## 3 Results

### 3.1 Nine suspected drugs were screened in stage 1

A total of 652 drugs were initially screened in stage 1. After combining drugs with the same ingredients and anatomical therapeutic chemical (ATC) classification, 346 drugs remained. After excluding external drugs and solvents for intravenous infusions (such as normal saline and glucose injection), 48 drugs were left (Supplementary Table S4). According to the inclusion criteria (a/b > 0.10 and b > 2000), a total of nine suspected drugs (diazepam, vitamin K1, cefamandole, omeprazole, ondansetron, ibuprofen, methotrexate, creatine phosphate sodium, and cytarabine) were finally identified. More information is shown in Table 1.

**TABLE 1:** Suspected drugs related to DIKI in pediatrics in stage 1.

Drug name	Pharmacological classification	ATC code	Number of DIKI events (a)	Total number of drug usages (b)	Ratio (a/b)
Diazepam	Sedative–hypnotic drugs	N05BA01	889	4,016	0.22
Vitamin K1	Vitamins	B02BA01	538	2,661	0.20
Cefamandole	Cephalosporins	J01DC03	1,028	5,212	0.20
Omeprazole	Mucosal protective agents	A02BC01	792	4,817	0.16
Ondansetron	Antiemetics	A04AA01	836	6,501	0.13
Ibuprofen	Antipyretics	M01AE01	2,313	18,990	0.12
Methotrexate	Antineoplastic agents	L04AX03	1,066	9,377	0.11
Creatine phosphate sodium	Cardioprotective drugs	NA	2,875	25,485	0.11
Cytarabine	Antineoplastic agents	L01BC01	881	8,343	0.11

Abbreviations: DIKI: drug-induced kidney injury; ATC: anatomical therapeutic chemical classification.

### 3.2 Six positive DIKI signals were identified in stage 2

**FIGURE 2:**
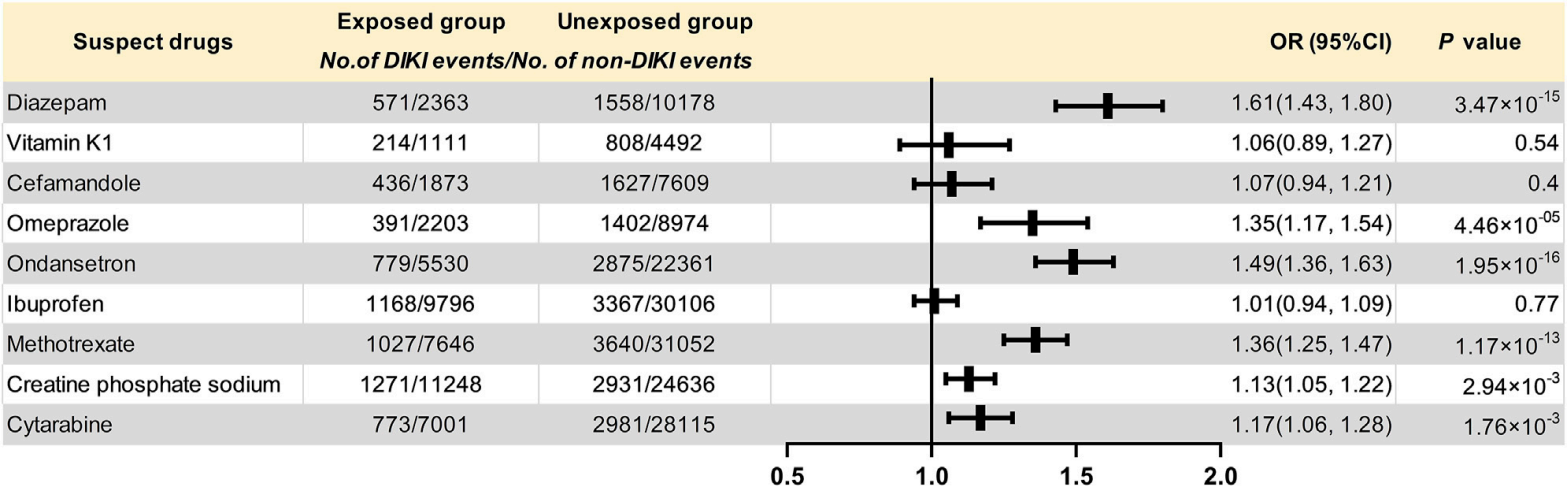
Forest plot for the association of suspected drugs with DIKI. Abbreviations: DIKI: drug-induced kidney injury; OR: odds ratio; CI: confidence interval.

The data extraction workflow of the nine suspected drugs is shown in Supplementary Table S5. The clinical information between the exposed group and the unexposed group is described in Supplementary Table S6. Retrospective cohort analysis showed that nine drugs met the inclusion criteria. Six drugs, namely, diazepam (*p* = 3.47 × 10^–15^, OR = 1.61, and 95% CI: 1.43–1.80), omeprazole (*p* = 4.46 × 10^–5^, OR = 1.35, and 95% CI: 1.17–1.54), ondansetron (*p* = 1.95 × 10^–16^, OR = 1.49, and 95% CI: 1.36–1.63), methotrexate (*p* = 1.17 × 10^–13^, OR = 1.36, and 95% CI: 1.25–1.47), creatine phosphate sodium (*p* = 2.94 × 10^–3^, OR = 1.13, and 95% CI: 1.05–1.22), and cytarabine (*p* = 1.76 × 10^–3^, OR = 1.17, and 95% CI: 1.06–1.28), were found to be associated with DIKI as positive signals. Although the three remaining drugs, namely, vitamin K1 (*p* = 0.54, OR = 1.06, and 95% CI: 0.89–1.27), cefamandole (*p* = 0.40, OR = 1.07, and 95% CI: 0.94–1.21), and ibuprofen (*p* = 0.77, OR = 1.01, and 95% CI: 0.94–1.09), tended toward a positive association with kidney injury, it did not reach statistical significance. The results of nine drugs and their associations with DIKI are shown in Table 2 and Figure 2.

**TABLE 2:** Signal detection of drug-induced kidney injury using PS matching in stage 2.

Suspect drug	Exposed group		Unexposed group		Adjusted *p*-value^a^	Or (95% CI)
	Number of cases with DIKI events	Number of cases without DIKI events	Number of cases with DIKI events	Number of cases without DIKI events		
Diazepam	571	2,363	1,558	10,178	3.47 × 10^–15^	1.61 (1.43, 1.80)
Vitamin K1	214	1,111	808	4,492	0.54	1.06 (0.89, 1.27)
Cefamandole	436	1873	1,627	7,609	0.40	1.07 (0.94, 1.21)
Omeprazole	391	2,203	1,402	8,974	4.46 × 10^–05^	1.35 (1.17, 1.54)
Ondansetron	779	5,530	2,875	22,361	1.95 × 10^–16^	1.49 (1.36, 1.63)
Ibuprofen	1,168	9,796	3,367	30,106	0.77	1.01 (0.94, 1.09)
Methotrexate	1,027	7,646	3,640	31,052	1.17 × 10^–13^	1.36 (1.25, 1.47)
Creatine phosphate sodium	1,271	11,248	2,931	24,636	2.94 × 10^–3^	1.13 (1.05, 1.22)
Cytarabine	773	7,001	2,981	28,115	1.76 × 10^–3^	1.17 (1.06, 1.28)

a : The *p*-value was adjusted by the Benjamini–Hochberg method.

### 3.3 Four new signals for DIKI in pediatrics were evaluated

According to the current available knowledge, the novelty of the six positive DIKI signals observed in stage 2 was further evaluated (Table 3). Of these, creatine phosphate sodium was found to be a type I signal as it had not previously been reported in either adults or children. Three other drugs, namely, diazepam, omeprazole, and ondansetron, were demonstrated to be type II signals in pediatrics. The other drug–DIKI associations have been confirmed in previous studies.

**TABLE 3:** Evaluation of drug-induced kidney injury signals.

Suspect drug	Literature (PubMed/Embase)		Literature (CNKI/Wangfang)^a^		SPC^b^	Signal type*
	Adults	Children	Adults	Children		
Diazepam	**√**	**×**	**×**	**×**	**×**	II
Omeprazole	**√**	**×**	**√**	**×**	**√**	II
Ondansetron	**√**	**×**	**×**	**×**	**×**	II
Methotrexate	**√**	**√**	**√**	**√**	**√**	Known
Creatine phosphate sodium	**×**	**×**	**×**	**×**	**√**	I
Cytarabine	**√**	**×**	**√**	**√**	**√**	Known

AbbreviationsDIKI: drug-induced kidney injury; SPCs: summary of product characteristics.

a : Literature reviewed: 1) PubMed: https://pubmed.ncbi.nlm.nih.gov; 2) Embase: https://www.embase.com; 3) Wanfang: http://www.wanfangdata.com.cn/index.html); and 4) CNKI: https://www.cnki.net.

b : SPCs reviewed: 1) Micromedex: https://www.ibm.com/watson-health/learn/micromedex); 2) FDA website: https://www.fda.gov/; and 3) drug instructions: https://www.yaozh.com/.

*Signal type **I**: the specific drug-DIKI signal had never been reported in the literature; **II**: the specific drug-DIKI signal had been reported in the literature about adults, but no reports about children could be found in the literature; known: the specific drug-DIKI association had been reported in both adults and children.

## 4 Discussion

Medications are the relatively common cause of AKI, especially in pediatrics. DIKI is related to a drugs’ inherent toxicity and how the kidneys are able to handle it. It poses several challenges for clinicians mainly because of the difficulties in timely identification of drugs with potential nephrotoxicity (Starr et al., 2021). In this context, research on RWD is growing for its implication in the surveillance of drug safety. The EHR, as one of the routine RWDs, is a good data source for pharmacovigilance because of its detailed information on clinical events related to medications (Patadia et al., 2015). This study established and applied a two-stage method for detecting DIKI signals in children using an EHR database: first to identify potential suspected drugs and then to conduct a retrospective cohort to analyze the correlation between the specific suspected drugs and renal impairment. We initially discovered nine candidate drugs related to DIKI, and six positive signals for DIKI were identified in further analyses. To the best of our knowledge, one of the drug-DIKI associations (creatine phosphate sodium) has not been previously described in current literature evidence, either in adults or in children. Three other drug-DIKI associations (diazepam, omeprazole, and ondansetron) were not previously reported in children but have already been reported in adults. These drugs may become the critical target drugs for active surveillance and causality assessment in further research.

### 4.1 Potential new signals

The association of creatine phosphate sodium with DIKI was found to be a possible new signal in this study. Creatine phosphate sodium is one of the cardioprotective drugs and is widely used in the protection of abnormal myocardial metabolism during myocardial ischemia. A previous study showed that 14.40% cases (28/200) of children were treated with sodium creatine phosphate as prophylactic treatment without a diagnosis, suggesting that there was an overdosage of sodium creatine phosphate in pediatrics (Jin et al., 2022). Another study showed that high doses of creatine phosphate sodium injection may cause large amounts of phosphate intake, which may affect renal function through calcium and purine metabolism disorders, as well as unstable hormone secretion (Yan et al., 2011). Recently, the National Medical Products Administration (NMPA) in China has recommended that serum calcium, serum phosphorus, and renal function should be monitored during the use of creatine phosphate sodium in neonates and premature infants, which also provides a reference for children of other ages. Although creatine phosphate sodium has been on the market for many years, the mechanisms underlying the association between its use and the deterioration of kidney function are still unclear. In light of these findings, further studies should assess the mechanism of kidney injury induced by creatine phosphate sodium in children. For patients with DIKI, suspected drugs were discontinued immediately, followed by symptomatic treatment to preserve the kidney. At the same time, clinicians should also pay attention to regular renal function in children who need long-term and high-dose use of creatine phosphate sodium to better promote the rational use of this drug.

Three other drug-DIKI associations (diazepam, omeprazole, and ondansetron) were identified as potentially new signals in the pediatric population. Diazepam is a common sedative–hypnotic drug in clinical settings, and the ADRs described in SPC include drowsiness, fatigue, muscle weakness, urinary retention, or incontinence. A nationwide population-based retrospective cohort study showed that diazepam had a significant correlation with increased chronic kidney disease risk in the population aged >18 years old (adjusted hazard ratio (HR) = 1.627, 95 CI%:1.527–1.736) (Liao et al., 2020). One of the possible mechanisms is that propylene glycol is contained in parenteral formulations of diazepam, which may cause AKI and proximal tubule injury (Cawley, 2001). In addition, omeprazole is a proton pump inhibitor (PPI) that selectively inhibits the H + -K + -ATPase in the gastric parietal cell membrane. It is widely used in the treatment of peptic ulcers, reflux esophagitis, and Zollinger–Ehrlich syndrome in pediatrics (Kato et al., 2021). Serious ADRs such as acute interstitial nephritis (AIN) and renal failure have also been reported in adults (Nadri and Althaf, 2014). Simpson et al. (2006) reported that the incidence of AIN caused by omeprazole was 8/100,000 (95% Cl: 2.6/100,000 to 18.7/100,000) (Simpson et al., 2006). Other studies have shown that omeprazole can cause a high incidence of renal damage in the elderly (Klatte et al., 2017). The underlying mechanism was drug-induced immune damage, including acute and chronic interstitial nephritis and tubulointerstitial nephritis (Torlot and Whitehead, 2016). Furthermore, ondansetron is a preferred anti-emetic in critical care to treat nausea and vomiting. A recent pharmacoepidemiology study reported that ondansetron may be associated with an increased risk of AKI (Gray et al., 2022). Another work showed that ondansetron can enhance cisplatin-induced nephrotoxicity *via* inhibition of multiple toxins and extrusion proteins (Li et al., 2013). However, the associations between the aforementioned three drugs and renal impairment were mainly reported in adults. We should also remember that most of the drugs currently used in pediatric clinical practices were originally developed for adults, and for the majority of them, the mechanism of renal toxicity in children is still to be clarified. Our results may provide more clues and need to be validated in a larger pediatric population.

This study also found that both methotrexate (MTX) and cytarabine were associated with a degree of kidney injury, which was consistent with previous reports in the literature. They are both common antineoplastic agents and are widely used in acute lymphoblastic leukemia (ALL) and non-Hodgkin’s lymphoma in pediatrics. A retrospective analysis found that the first high-dose methotrexate (HDMTX) course (OR = 1.767) and methotrexate dose per body surface area (OR = 1.944) significantly correlated with AKI in 336 ALL children (Cheng et al., 2018). HDMTX-associated AKI with delayed MTX clearance has been linked to an excess in MTX-induced toxicities (Heuschkel et al., 2022). AKI develops due to the precipitation of MTX and its metabolites within the tubular lumens of the kidney (Perazella and Izzedine, 2015). Furthermore, MTX has been shown to induce the formation of oxygen radicals with subsequent cellular injury, associated with decreased adenosine deaminase activity (Pinheiro et al., 2010). In addition, a case report showed that low-dose cytarabine could induce hepatic and renal dysfunction in a patient with myelodysplastic syndrome, indicating that careful observation should be carried out in clinical practice (Tanaka et al., 1999). As an anti-tumor drug with weak cell specificity, cytarabine affects all actively growing cells by inhibiting the synthesis of DNA, including renal tubular epithelial cells, thereby causing nephrotoxicity (Pannu and Nadim, 2008). The aforementioned results support the reliability of our two-stage method in DIKI signal detection.

### 4.2 Strengths and limitations

The main strength of this study is its capability to retrospectively observe a large number of children and adolescents in a “real-world” setting by combining data from longitudinal EHRs. This two-stage designed approach has certain advantages in comparison with other methods based on abnormal values of laboratory tests (Yoon et al., 2012) or GTT. In the first stage, we roughly assessed the potential to select the suspected drugs, increasing the efficiency of subsequent analysis. Furthermore, more confounders, such as kidney disease and medications that may affect the level of laboratory indicators, were considered. In addition, the algorithm execution does not require manual review and can be developed into an automated program in the future, which is more suitable for the detection of ADEs based on large-scale databases. The final results suggest that our method can be used as a useful tool to detect DIKI signals for use by clinicians and regulatory agencies.

Despite the strengths of EHRs, they have inherent limitations, including incomplete case capture, selection bias, unmeasured confounding, and the inability to infer causality. Thus, some limitations to this study should be considered. First, only the records that met the criteria were included in the study. The associations between suspected drugs and DIKI remain unknown in those excluded records. Second, while our method has controlled four important variables, it does not adjust drug–drug interactions, drug–dose response, or disease severity covariates in the model. Third, the EHR data from a single-site system was used in this study. More efforts are needed to validate the feasibility of the approach and the DIKI signals in other EHR databases in the future. Lastly, it is important to realize that this method is a tool to assist with signal detection but does not ensure causality assessment of ADRs. All potentially new signals require further evaluation in hypothesis-testing studies to better account for bias and confounding.

In summary, this study shows that the RWDs, especially the EHRs, are potentially valuable resources for post-marketing drug safety surveillance. Our findings provide six candidate drug-DIKI associations using medical record databases. Future research is warranted to assess the causality of DIKI signals and formulate strategies for drug risk management in pediatrics. Nowadays, China has initiated the project “China ADR Sentinel Surveillance Alliance” (CASSA) with at least 300 medical facilities. Also of note, BCH was the first pediatric hospital in CASSA. More attention will be paid to integrating the method based on the CASSA platform to explore multi-center pharmacovigilance research.

## 5 Conclusion

DIKI is one of the most common problems in clinical practice, especially in hospitalized patients. In this work, we proposed a quantitative two-stage procedure to explore potential DIKI signals using RWD. Six positive signals of DIKI, including four new signals in children, were detected. Our findings highlight the potential of EHRs to complement traditional SRS for drug safety signal detection and strengthening in a pediatric setting. It is hoped that current efforts to identify DIKI signals will allow us to modify practice and reduce unnecessary harm in the future.

## Data Availability

The raw data supporting the conclusion of this article will be made available by the authors, without undue reservation.
